# Meetings of Societies

**Published:** 1898-06

**Authors:** 


### MEETINGS OF SOCIETIES.

### Bristol Medico-Chirurgical Society.

*March 8th*, 1898.

Dr. J. E. Shaw, President, in the Chair.

Considerable time was occupied in discussing the propriety of the insertion of medical autobiographies in a lay publication.

Dr. Michell Clarke showed (1) a case of paralysis agitans at an unusually early age. The patient was a man aged 38, and the symptoms first appeared as occasional attacks of tremor—soon becoming constant—in the left arm. Both sides of the body are now affected, the right since three months. (2) A man affected with multiple neuritis of alcoholic origin. The case was a very severe one, of twelve months' duration. The disease began in the legs and spread to the arms. The interesting feature was the wasting of the small muscles of the hands that had occurred. In both arms and legs the muscles were symmetrically affected. The patient was now beginning to recover.

Mr. T. Carwardine exhibited some microscopical specimens of implantation cysts, and referred to an original illustration of an implantation cyst of the thumb, and to an illustration by Barker of a microscopic section of an implantation cyst in the Pathological Society's *Transactions*. Implantation cysts are due to proliferation of epithelium driven into the subcutaneous tissues by some traumatism, accidental or surgical. Accordingly, they occur in parts most liable to injury. Of twenty-one cases of cysts of the iris recorded by Hulke in the Reports of the Royal London Ophthalmic Hospital for 1869, seventeen cases were the result of injury. They occur frequently on the fingers, particularly the palmar surfaces of the terminal phalanges of the thumb and forefinger. Occasionally they are found in the palm of the hand. Animals are not exempt, for they are found in the axillæ of oxen which have been "prodded," and in animals injured by spikes and barbs of fences. The first microscopic specimen showed a minute and complete implantation cyst with a diverticulum leading to a sebaceous gland. The patient was a woman who was injured five months previously on the side of the nose by a stone, and she noticed a small lump for the first time five weeks before admission. The small tumour looked like an inflamed sebaceous cyst, and the contents also resembled such a tumour; but on very careful microscopical examination a small epithelium-lined cyst was found resembling inverted skin. The second specimen was from an implantation cyst of the arm. It showed trabeculæ with compressed papillæ of cutaneous epithelium, and cells like those of the Malpighian layer next to the trabeculæ. The third specimen was from a tumour of the finger which was recently removed by Mr. Munro Smith. The tumour was of thirty years' duration. Although no history was obtained, Mr. Carwardine regarded the microscopical appearances as characteristic of an implantation cyst.—Mr. C. A. Morton referred to a case of this kind he had published a few years ago.[1] The epithelium had been carried into the deeper tissues by a thorn, several years before the cyst was noticed. It contained a black mass, which on soaking in alcohol split up into layers of corneous epithelium.—Mr. F. R. Cross mentioned a case[2] under his care where a cyst on the iris followed a wound of the cornea with wire which carried an eyelash into the anterior chamber. The eyelash was removed with some of the adjoining iris; but active cells of the lash remained, and an implantation cyst lined by laminated epithelium was the result, as shown later by microscopical examination of the enucleated eyeball. He also published[3] the case of an implantation cyst following a good extraction of cataract, which gave excellent vision for several months. Glaucoma supervened. Enucleation of the eyeball gave the opportunity of microscopic examination, which exposed a typical implantation cyst lined by laminated epithelium.

Mr. C. A. Morton showed a large tumour weighing 6½ lb. which he had removed with the breast from a woman aged 51. It had been growing for nine years, but had doubled its size in the two months before removal, and had then become very painful. Before removal

1 *Bristol M.-Chir. J.*, 1894, xii. 233. 2 *Tr. Ophth. Soc. Lond.*, 1893, xiii. 199.
3 *Ibid.*, 1892, xii, 175, and *Brit. M. J.*, 1892, ii. 130.

it presented some very soft bosses with stretched and purple skin over them; but the greater part of the growth was very hard, and many nodules movable on the large mass were detected. The tumour was of much interest, not only on account of its great size, but also because of its structure. The great mass of the growth was the ordinary fibro-adenoma; but here and there were nodules of tissue perfectly defined from the rest, and quite different both in naked-eye and microscopic structure. They were much softer than the fibro-adenomatous portions, of a yellow colour, and histologically showed a structure of small round cells, with a few oval and spiral cells, but no gland tissue whatever. Hence the tumour was a combination of pure sarcoma with fibro-adenoma. —Mr. J. LACY FIRTH remarked that these benign tumours termed fibro-adenomata may have the inter-acinous tissue either adult fibrous tissue or embryonic tissue; but some authorities did not approve that the term sarcoma should be applied to these cases, for there was no evidence that clinically they behaved as malignant tumours.—Dr. AUST LAWRENCE drew attention to the occasional combination of fibroid uterus and sarcoma, showing the analogy of the uterus and breast.—In reply, Mr. MORTON said that he had not spoken of the tumour structure to which Dr. Firth evidently referred—adeno-sarcoma of the breast. It was not a combination of this tissue with fibro-adenoma—a mixture common enough—but the presence of nodules of pure sarcoma without any gland tissue, in an enormous fibro-adenoma, that was so interesting. He would not expect the glands to be affected in sarcoma of the breast, and it was too early to say whether metastatic growths were likely to occur or not. In connection with the subject to which Dr. Lawrence had called attention—the similar combination of sarcoma with fibro-myoma of the uterus, or rather the supposed transformation of the innocent into the malignant growth—Mr. Morton called attention to a recent statement of Mr. Bland Sutton, that it was desirable to have evidence of secondary growths in cases in which such transformation was said to occur. In Dr. Lawrence's case he had found at the necropsy secondary growths in all parts.

---

*April 13th*, 1898.

Dr. J. E. SHAW, President, in the Chair.

Mr. PAUL BUSH, for Mr. C. A. Morton, showed a patient from whom Mr. Morton had excised the upper end of the tibia for myeloid sarcoma, and who was exhibited at a meeting of the Society a year ago.[1] The president of the Society had requested Mr. Morton to show the patient again in a year's time. There was no sign of recurrence, nor was such to be expected, as the growth was myeloid; and the man was able to get about and attend to his work very well. The shortening of the limb was made up for by a high-soled boot. No tendency to flexion had been observed, the union being osseous. Mr. Morton had operated on another case of myeloid sarcoma in the head of the tibia in the same way since operating on the case shown; both cases will shortly be published.

Mr. F. LACE showed a specimen of duct papilloma of the breast which he had removed from a girl aged 16. He remarked that the earliest recorded age at which such a condition had been found was 23. The tumour occupied the usual central position,

[1] *Bristol M.-Chir. J.*, 1897, xv. 212.

with elastic prominences around the nipple, which was inverted, as was also the other nipple. There was some bloody discharge from the nipple. The tumour resembled exactly the condition figured by Bland Sutton, and which he calls intra-cystic papilloma, which occurs in the lacteal sinuses. Mr. Lace drew attention to the microscopical structure, which was unlike any illustration he had seen, in that, instead of the fibrous framework being scanty with scarcely any cellular elements, in this case the cells were very numerous, as were also the blood-vessels, the appearances being very suggestive of sarcoma.

Mr. Paul Bush showed (1) a tumour of the ovary weighing $4\frac{1}{2}$ lbs., which he had successfully removed from an elderly lady during an acute attack of peritonitis, due to twisting of the pedicle. The tumour had been noticed for about ten years, and during the last two months had caused increasing obstruction to the bowels. On opening the abdomen a large quantity of bloody peritoneal fluid escaped, and the surface of the tumour showed decided signs of sloughing commencing: he exhibited numerous microscopical slides, which proved that the tumour was a fibro-myoma undergoing well-marked myxomatous degeneration, and he commented on the fact that this form of tumour of the ovary is not by any means a common one. (2) A large tumour of the vertebræ: it occurred in a young girl. At the *post-mortem* examination no other primary growth could be found, although every organ of the body was carefully examined; the microscopic slides demonstrated that it was a carcinoma and not a sarcoma.—Mr. Munro Smith considered that the tumour of the ovary shown by Mr. Bush consisted of two distinct structures, one myxomatous and the other myomatous, *i.e.* consisting of unstriped muscular fibres.—Dr. Watson Williams made some remarks on the question of so-called myxomatous degeneration. He referred to the fact that nearly all "mucous polypi" of the nose were really œdematous fibromata, and asked whether in Mr. Bush's case and in other similar cases the myxomatous degenerations might not be œdematous fibroma tissue rather than true myxomatous tissue. He suggested that the twisting of the pedicle might be one cause of such infiltration, producing, as it would, obstruction to the venous and lymphatic circulation.—Dr. Aust Lawrence thought the specimen to be one of fibroma of ovary, most likely showing slow degeneration with hemorrhage.—Dr. Stack thought the appearance unlike myxoma of the nose, the cells being so much more closely set; and that the myxomatous part was probably such *ab initio*, and that the specimen should be regarded as myxo-fibro-myoma, and not a myxo-fibro-myo-sarcoma.

Dr. J. O. Symes exhibited three plates from different glycerinated calf-lymphs. All showed colonies of staphylococci and bacilli, the number of colonies being approximately proportional to the cloudiness of the lymph; one of the plates developed colonies of streptococci. One plate of unglycerinated human lymph, nearly clear, developed only three or four colonies of staphylococci and bacilli. The point of chief importance is to obtain a clear transparent lymph.—Mr. J. Ewens in his practice had never seen any bad results, when reasonable care as regards cleanliness, and ordinary sanitary precautions, had been taken. He preferred human to calf lymph.

Dr. Theodore Fisher showed a selection of pathological specimens from those added to the Museum of the Bristol Royal Infirmary during the past year. There were, amongst others, the following specimens: A case of thrombosis of the basilar artery in diphtheria; Thrombosis of the meningeal veins in noma; Tricuspid stenosis; Tubercular disease of the pericardium; Subphrenic abscess opening into the left lung; Fibroid disease of the lung, secondary to the presence of a portion of a tracheotomy tube in the bronchus; Thrombosis of the portal vein; Small chronic tropical abscess of the liver; Rupture of the pancreas; Abscess of the head of the pancreas; Adenomata of the kidney.

Dr. B. M. H. Rogers read a paper on "Isolation after Diphtheria." (For this and the discussion which followed, see pp. 101–108.)

Mr. James Taylor showed a lantern-slide of the temperature charts of twenty-three cases of influenza occurring in a girls' orphanage of forty-four inmates. The cases all occurred between January 12th and January 29th, 1898. In eleven of the cases after the initial fever, the rise of temperature continued for fourteen days or more, without any apparent complication. The rise was slight—about 99°—and in most of the cases it occurred in the morning, going down to normal in the evening. It did not seem due to the onset of menstruation, as some of the children were only eight or ten years old. There was an utter absence of any bronchial complications in all the cases. Three or four had slight tonsillitis. One child had enlarged glands in the neck which threatened to suppurate. In another case on the fourteenth day the temperature suddenly ran up to 104°, for which no cause could be found. As it subsided in thirty-six hours and the child made an uneventful recovery, it was thought to be a recrudescence of the influenza. Vomiting on the sixth day, lasting for twenty-four hours, occurred in one case, with no abdominal symptoms whatever, thought to be due to the influenza poison affecting the vomiting centre.—Dr. Markham Skerritt said that the interesting series of cases brought forward was in accordance with what was probably the usual experience—that while at its first appearance influenza had occurred in attacks of severe fever of short duration, in its later outbreaks the type had altered, so that the febrile symptoms were less intense and more prolonged. In his experience no drug had the slightest effect in cutting short the elevation of temperature.

Mr. Munro Smith showed some lantern slides illustrating the development of the embryo.

---

*May 11th*, 1898.

Dr. J. E. Shaw, President, in the Chair.

Dr. A. J. Harrison and Mr. G. Munro Smith were appointed Auditors for the Society's accounts.

Dr. Barclay Baron showed a man, 68 years of age, who never remembers having breathed properly through his nose, and at 8 years old he was under treatment for ulceration of the nose. He has practically no anterior opening through the left nostril, and a very small one through the right nostril. The uvula is gone, the soft palate and pharynx are extensively cicatrised. On examining the naso-pharynx with a mirror, the septum is visible; there is no opening through the left nostril, it being occluded by a web, probably due to cicatricial tissue formation. On the right side there is an opening about the size of a pea. The case is interesting from its showing the extreme form of the ravages of syphilis of congenital origin.

Dr. J. O. Symes showed specimens of lepothrix (trichomycosis nodosa) showing fine bacilli or oval cocci. The condition was accompanied by red sweat, and a red pigment-forming organism was isolated.

Dr. W. H. C. Newnham showed a dermoid cyst successfully removed from E. N., aged 35, who was admitted to the Bristol General Hospital on March 2nd, 1898, with large abdominal tumour and great pain in right iliac region. On April 10th the tumour was removed from the right ovary. It contained a tuft of hair, some cartilage, a large bony plate, and a quantity of sebaceous material. The patient made an uninterrupted recovery.

Dr. W. H. C. Newnham read a paper on "Twelve Abdominal Sections." (1) *Disease of Ovaries* (*Cystic*). E. D., aged 42, was sent in April, 1897, for disease of both ovaries. The abdomen was opened and two large cystic ovaries were removed. The operation was completed in twenty minutes. The patient recovered without a bad symptom, and left one month after the operation. She is now in very good health, and has not suffered in any way since the operation. (2) *Ovarian Cyst.* On April 27th, 1897, a patient of Dr. Belfield was suffering most intense abdominal pain. She had kept her bed for some weeks. On opening the abdomen a semi-solid cyst, which turned out to be sarcomatous growing from the right ovary, was found. There were many adhesions, some to the intestine, which were separated with great difficulty. The operation lasted thirty-five minutes, and the patient made a very slow recovery, but she is now apparently quite well. (3) *Sarcomatous Tumour of Ovary.* M. T., aged 25, was admitted to the Bristol General Hospital on May 28th, 1897. While waiting for a consultation, patient was suddenly taken with symptoms of acute peritonitis. At 8.30 p.m. a very large solid sarcomatous tumour of the right ovary was removed. Posteriorly some of the small cysts had given way and were leaking into the general peritoneal cavity; this was apparently the cause of the peritonitis. The abdomen was thoroughly washed out and drained, the whole operation lasting thirty minutes. The patient recovered completely, but did not leave the Hospital until August 6th. How soon this may return, or whether it will at all, of course it is impossible to say. At present she is very well. (4) *Ovarian Tumour.* E. S., aged 50, sent up from Wales. She had noticed the swelling of her abdomen for two years. It was diagnosed as a simple ovarian cyst, and on July 27th it was removed. A gallon and a half of clear fluid ran from the cyst. The operation lasted thirteen minutes, and the patient left the Hospital well on August 21st. (5) *Ruptured Tubal Pregnancy.* M. G., aged 37, was first seen in consultation. She was supposed to have miscarried at four or five months. The uterus was empty, but there was a very large swelling in the pouch of Douglas. She was removed to the Hospital, and on August 13th, 1897, the abdomen was opened and a male fœtus and placenta were removed from Douglas's pouch. It was about the fourth to fifth month. The sac was thoroughly washed out and drained. The operation lasted thirty-five minutes. She went on well for a week, and then suddenly became quite changed, and died on the ninth day after the operation. At the necropsy general peritonitis was found. (6) *Ovarian Cyst.* L. C., aged 57, was admitted August 27th, 1897. On September 2nd, after consultation, the abdomen was opened and a very large ovarian tumour growing from the left side was found. When the cyst was emptied very numerous and dense adhesions to the intestine of the cyst-wall were found, and there was great difficulty in separating them. It was thought best to drain. The operation lasted fifty-three minutes. She was convalescent in one week, and left the hospital very shortly quite well. (7) *Pelvic Hæmatocele.* E. H., aged 24, admitted September 2nd. On September 11th the abdomen was opened, and a large quantity of dark clot (no placental-like tissue or trace of fœtus discovered) removed from a sac. This was thoroughly washed out with boracic lotion and drained. The operation lasted seventeen minutes. She had no bad symptoms, and left the hospital well on October 14th. (8) *Uterine fibroids.* L. P., aged 30, sent by Dr. Martin, of Westonsuper-Mare, for enormous fibroids, with excessive continuous uterine hemorrhage. After consultation, it was decided to open the abdomen and be guided by the condition of things then found. At the operation on January 18th, 1898, it was found that the simplest proceeding would be to remove two large cystic ovaries. This was done; but after ligaturing in the usual way, first the ligature on the right side and then that on the left slipped. After some trouble all the vessels were tied separately. After this no further trouble arose; there was no further uterine hemorrhage, and the patient made a very good recovery, leaving the hospital on February 15th. (9) *Ovarian Tumour.* M. P., aged 23, sent to me on January 23rd, 1898, by Dr. Norton. She had noticed a tumour in the abdomen for about a year. The diagnosis was an ordinary ovarian tumour on the right side. The abdomen was opened on February 1st; there were no adhesions, and the operation was apparently completed in ten minutes. As some hemorrhage was going on, the pedicle was pulled up, and it was found that the ligature had slipped. It was again transfixed and tightly tied, and a drainage-tube now inserted on account of the blood in the peritoneal cavity. In the evening the house-surgeon sent, as seven ounces of blood had been sucked up from the tube; but as the patient appeared so well and had such a good pulse, it was decided to leave her alone. She left the hospital quite well on February 27th, not having had a single bad symptom since the night of the operation. (10) *Malignant Disease of Omentum.* M. C., aged 38 years, was seen on January 23rd, 1898, with tumour of abdomen and vomiting. There was no trustworthy history; but the tumour itself appeared to be freely movable, with some fluid in the peritoneal cavity—an irregular part of the tumour, or what appeared like it, was to be felt *per vaginam* behind the uterus. It was also distinctly felt *per rectum.* Four days later the abdomen was opened. There was a large quantity of ascitic fluid, with very extensive malignant disease of the omentum; the peritoneum was also studded. A drainage-tube was immediately put in. The after-history up to the present has been uneventful: the patient returned home in a fortnight, apparently much better; the sickness had then quite stopped, and she is now better than before the operation. (11) *Pyo-salpinx.* A. R., aged 45, admitted to the Hospital for swelling of abdomen, supposed to have lasted for eight days, coming on with sudden faintness, sickness, and some uterine hemorrhage. On admission she had great tenderness of the abdomen, more especially in the right iliac region, and at this spot some amount of thickening and resistance could be made out. She was so very anæmic, and as this was markedly increasing, it was thought that some hemorrhage might be taking place into the peritoneal cavity. On February 16th, 1898, the day after admission, a consultation was held, and the same afternoon the abdomen was opened, and mischief connected with the right ovary and tube was found; and, after unusual difficulty in separating the adhesions, the ovary and an enormously dilated tube containing some purulent matter were removed. The tube was with great difficulty distinguished from a piece of intestine. The abdominal cavity was irrigated with saline solution, and a large quantity left behind. There was some caseating material in a portion of the tube. Patient now doing very well. Wound healed. Drainage-tube remained in for a week. (12) *Ovarian Cyst.* E. P., aged 30. Confined seven months ago, and then noticed she still remained about the size of pregnancy at six months. She was sent by Dr. Walker, of Coleford, on February 8th, 1898. On February 18th the abdomen was opened, and an ovarian cyst found, containing about ten pints of dark-brown fluid, with the pedicle so twisted that at least six complete turns were required to straighten it out. No drainage-tube was used, and the operation only lasted thirteen minutes. The stitches were all removed on the tenth day, and patient is now quite well.—Mr. C. A. MORTON said that since the beginning of 1897 he had performed abdominal section in thirty-six cases. In this time all his operations for ovarian and uterine tumours, for gallstones, and for recurrent appendicitis had been successful. He had gained courage in dealing with abscesses lying deeply in the pelvis, and had operated successfully on three such cases by evacuating the pus through the peritoneal cavity, thoroughly flushing with sterilised saline solution and draining. The last abdominal case on which he had operated was sent to him as one of ovarian cyst. All that could be made out was a very soft fluctuating area in the right lower abdomen. It had no definite outline, and could hardly be called a swelling. It turned out to be a cyst in the right broad ligament, raising the pelvic peritoneum over it, and lying in contact with the iliac vessels and ureter, and the side of the uterus. Its wall was very lax and soft, and it required much careful dissection for its removal. The result was quite satisfactory.—Mr. T. CARWARDINE said it was important to know why there was slipping of ligatures in the cases mentioned.—Dr. E. H. E. STACK would like to know the result of microscopic examination of the two cases of sarcoma, and whether the opposite ovaries were normal. —In reply, Dr. NEWNHAM said that the silk used in the three cases where the pedicle slipped had possibly been boiled too much. He now used silk kept in a mixture of carbolic and spirit. A microscopic examination was made of the sarcomatous tumour removed; there was no doubt about it: at the time of the operation the other ovary appeared to be quite healthy.

Mr. W. R. ACKLAND read a paper on "Some Secondary Effects of Dental Irritation." In his own experience he had seen a boy who was myopic, and in some degree astigmatic, get infinitely better when his mouth, which had been rather overcrowded, was put into good order. In another case there was photophobia and discolouration of the iris, with a slight decrease of ocular tension of the left eye. When two bicuspid stumps which showed signs of tenderness were removed, the photophobia disappeared, and the discolouration of the iris gradually cleared up. He had seen other cases in which photophobia was a concomitant of dental mischief. In a patient of middle age the extraction of the right lower wisdom tooth, which was decayed, was followed by the loss of earache and deafness from which the patient had suffered for some time. He had seen other cases, principally in children, in which otalgia depended upon dental irritation. He had also seen one or two cases of otorrhœa due to the same cause, and a case in which an obstinate chronic rhinitis disappeared after the extraction of a canine root; but this may have been a coincidence. Text-books and journals give a long list of all sorts of reflex troubles, even uterine, due to dental irritation, and it is well to have this possible cause always in view.—Mr. W. H. HARSANT had frequently met with cases of otalgia due to dental irritation, but was inclined to be sceptical as to any connection between middle-ear suppuration and carious teeth: he thought it was more probably a case of two distinct affections existing simultaneously.—Mr. EWENS remarked that neuralgic pain in the ear was frequently associated with decayed teeth in the lower jaw.—Dr. E. J. CAVE drew attention to referred pain in the upper arm and neighbourhood of the shoulder in connection with dental caries.—Dr. AUST LAWRENCE thought that the only relation between the teeth and the uterus was that there was an os connected with both.

Professor Fawcett exhibited (1) Various anatomical specimens and models, amongst which were: (*a*) A series of carpal and metacarpal bones, mounted separately on removable handles, after a method in use at Leeds; (*b*) A model in wax of the muscles, vessels, and nerves of the orbit, by Tramond of Paris. This model was presented to the anatomical museum by the President of the Bristol Medico-Chirurgical Society (Dr. J. E. Shaw); (*c*) A very successful cast in plaster of the right cerebral hemisphere.

(2) A series of sections (microscopical) of the hind and mid brain prepared by Campbell's modification of the Pal-Weigert method. These specimens were shown on the screen by the aid of the lantern, and the tract of the fillet was especially dealt with.

(3) A number of specimens of the frontal sinuses opened in various ways, showing especially the following points: (*a*) That the sinuses vary very much in size individually; (*b*) That they do not necessarily correspond with the elevations—superciliary ridges and glabella—under which they lie, several specimens showing large elevations and very small sinuses; (*c*) That they are usually asymmetrical, the asymmetry corresponding with that of the nasal cavities; (*d*) That the best site for trephining with certainty is in the region of the internal angular process of the frontal bone, as has been insisted upon by Mayo Collier, and Tilley (*Lancet*, 1896), and others; (*e*) That a perforation in such a situation is most likely to reach the particular sinus desired.

J. Paul Bush, *Hon. Sec.*

